# Media Reporting Relating to COVID-19 Vaccination as a Driver of Vaccine Hesitancy Prior to the Second Wave of the COVID-19 Pandemic in India: A Content Analysis of Newspaper and Digital Media Reports

**DOI:** 10.7759/cureus.36750

**Published:** 2023-03-27

**Authors:** Saurav Basu, Himanshi Sharma

**Affiliations:** 1 Indian Institute of Public Health - Delhi, Public Health Foundation of India, New Delhi, IND; 2 Faculty of Social Science, Jamia Millia Islamia, New Delhi, IND

**Keywords:** media reporting, covaxin tm, second wave of covid-19, covid-19 india, vaccine hesitancy india

## Abstract

Background

Over 2,40,000 deaths were attributed to the SARS-CoV-2 Delta (B.1.617.2) variant in India during the second wave of the pandemic from April to June 2021 with most deaths occurring in the unvaccinated population. High levels of coronavirus disease 2019 (COVID-19) vaccine hesitancy contributed to significantly reduced vaccination coverage in the eligible population especially among healthcare workers, comorbid and older people. The existing global evidence suggests misinformation through social media to accentuate, while newspaper and mainstream media reporting to be protective against vaccine hesitancy during the COVID-19 pandemic. Content analysis of regular press coverage of COVID-19 vaccination in India during the period of initial deployment and until the onset of the second wave of the pandemic can provide useful learnings and strengthen preparedness for addressing potential vaccine hesitancy concerns during future pandemics. Therefore, we conducted this inductive content analysis of press coverage related to the COVID-19 vaccine hesitancy in India prior to the second (Delta) wave of the COVID-19 pandemic.

Methods

We examined news reports related to COVID-19 vaccination in India for the period from 1st January 2021 to 28 February 2021 from a high circulation English language daily (Hindustan Times), Hindi (vernacular) language daily (Dainik Jagran), and English language news reports from selected digital news portals. The inclusion criterion was any news report related to COVID-19 vaccination including editorials and guest opinion pieces that could potentially generate COVID-19-related vaccine hesitancy. The news items were classified depending on their potential to drive vaccine hesitancy by either avoiding reporting of positive information related to COVID-19 vaccines, or attributing directly or indirectly, negative or misleading commentary relating to vaccine safety or efficacy. Reports with possible risk of increasing vaccine hesitancy were further analyzed based on content, source of information, and the extent of fact-checking.

Results

Most of the published newspaper reports examined in this study echoed official news sources and views from government health agencies promoting COVID-19 vaccine acceptance and dispelling doubts on concerns regarding vaccine safety. There were eight unique newspaper reports after excluding duplicated bilingual entries and four news items from online digital Indian news sources that met our criterion of reports with possible contribution to vaccine hesitancy.

The reports possibly contributed to vaccine hesitancy were grouped into two themes: (i) Doubts on the safety and efficacy of local manufactured vaccines: most of these reports focused on the granting of emergency use authorization for Covaxin (BBV152) in ‘clinical trial mode’ without the completion and publication of Phase-3 efficacy data (ii). Doubts on vaccine requirement considering high seroprevalence and reduced virus transmission.

Conclusions

Concerns about the efficacy and safety of Covaxin (BBV152), safety of the Covishield vaccine, and questioning the necessity of immunizing all adults with COVID-19 vaccines were observed in multiple press reports with attempts at politicization of vaccination-related decisions. The press reporting with potential for contributing to significant COVID-19 vaccine hesitancy since launch and until the Delta wave of the pandemic in India has important lessons in future pandemic preparedness.

## Introduction

India experienced a major second wave of the coronavirus disease 2019 (COVID-19) pandemic during April to June 2021, largely attributed to the circulation of the severe acute respiratory syndrome coronavirus 2 (SARS-CoV-2) Delta (B.1.617.2) variant with an estimated 2,40,000 deaths occurring in the country during this period [1]. Failure to vaccinate nearly 85-90% of the vulnerable adult population was a major factor driving the second wave of the COVID-19 pandemic in India for it is well-established that vaccination with most of the available COVID-19 vaccines significantly reduces the odds of severe disease, hospitalization, and death [2-4]. Vaccine hesitancy referring to the delay in the acceptance or refusal of vaccines despite their availability was a significant reason for the low uptake of COVID-19 vaccines in India prior to the Delta wave especially among eligible health workers, comorbid, and older people [5,6]. 

During the pandemic, positive news on the COVID-19 vaccine was found to positively influence COVID-19 vaccine acceptability in the exposed population while exposure to negative or misleading information on COVID-19 vaccines correlated with an increase in vaccine hesitancy [7]. The role of social media in mediating COVID-19-related vaccine hesitancy through misinformation especially related to vaccine efficacy or safety has been a widely reported phenomenon, globally [8-9]. Conversely, reporting by mainstream media has been usually considered to have had a positive influence in dispelling myths and concerns related to safety of vaccines and restoring public trust and confidence in them, in contrast to those reliant on social media for obtaining vaccine-related news updates [10]. For instance, an observational study from sub-Saharan Africa ascertained that COVID-19 vaccine hesitancy was significantly lower in newspaper readers compared to social media and television users [11]. Another cross-sectional survey in Ghana observed higher COVID-19 vaccine acceptability in users of traditional channels of information such as newspapers compared to either social or both social and traditional media users [12]. Nevertheless, the possible failure of mainstream media in preventing its use and misuse in driving vaccine hesitancy has not been sufficiently explored. Moreover, with the increasing digitization of media, selected news reports from mainstream media are also frequently circulated in social media which may blur distinctions between these divergent information channels. Furthermore, belief in the safety and efficacy of vaccines available domestically as compared to foreign vaccines in the local populations could also escalate vaccine hesitancy with a likely driver emerging from the phenomenon of ‘vaccine nationalism’ linked to the concept of ‘lexicalization’ (Van Dijk, 2000) [13]. Consequently, comparative media reports related to COVID-19 vaccines on examination were observed to be frequently aligned with nationalist discourse by proclaiming only “good and positive things about our vaccines” while refraining from stating any “bad and negative things about our vaccines”. In contrast, such reports focused on “bad and negative things about their vaccines” and avoided stating “any good and positive things about their vaccines” (Abbas, 2021) [14]. 

India’s COVID-19 vaccination campaign was the largest in the world and involved multiple steps from approval, information-education-communication campaigns, deployment of digital technology for vaccination management, training of thousands of health workers, etc. [15]. The emergency use authorization (EUA) for the COVID-19 vaccines in India were provided by the Subject Expert Committee (SEC) consisting of domain experts of the Central Drugs Standard Control Organization (CDSCO), the national regulatory body for pharmaceuticals and medical devices in the country. Early milestones were the EUA on January 2, 2021 for the ChAdOx1 nCoV-19 (Covishield; Serum Institute of India, Pune) equivalent to the Oxford AstraZeneca (AZD222) vaccine which is a recombinant adenoviral vector vaccine [16,17]. On January 3, 2021, ‘restricted emergency use in clinical trial mode in public interest with abundant precaution’ purportedly to provide more vaccination options against mutant strains was granted to BBV152 (Covaxin; Bharat Biotech International, Hyderabad) which is a whole virion inactivated vaccine. [16,18,19]. The launch of the campaign for COVID-19 vaccination in India for health and frontline workers occurred on January 16, 2021, with vaccination provided free of cost through government health facilities. On March 1, 2021, the COVID-19 vaccination campaign was extended in the general population to older people aged >60 years and those with selected comorbidities while from April 1, 2021, the program was extended to all people aged >45 years. In the same month the onset, spread, and rapid progression of the Delta wave of the COVID-19 pandemic in India was observed [20].

To date, no content analysis of regular press coverage of COVID-19 vaccination in India during the period of initial deployment and until the onset of the second wave of the pandemic has been conducted. It is important to critically understand the forces that can undermine vaccine confidence and promote vaccine hesitancy through content analysis of mainstream media coverage during the pandemic since it can provide useful learnings and strengthen preparedness for addressing potential vaccine hesitancy concerns during future pandemics. Furthermore, primary data collection, months or years after the event will be unable to capture the underlying reasons for COVID-19 vaccine hesitancy especially in absence of a holistic understanding of the sociopolitical environment that stimulated people to avoid government, administrative, and scientific recommendations for vaccination. Therefore, we conducted an inductive content analysis of press coverage related to the COVID-19 related vaccine hesitancy in India prior to the second (Delta) wave of the COVID-19 pandemic.

## Materials and methods

Data source and analysis: This qualitative descriptive content analysis examined news reports related to COVID-19 vaccination for the period from 1st January 2021 to 28 February 2021 from the following sources: 1. Hindustan Times (HT), English language daily newspaper; 2. Dainik Jagran (DJ), Hindi language daily newspaper; 3. Online (digital) news-reports published during the same time in web-based (digital) English language Indian news portals through google searches and from content shared in the author’s social networks (Facebook/Twitter/WhatsApp) that were identified as possibly influencing COVID-19 vaccine hesitancy. Both the newspapers were purposively selected due to their large circulation especially in Northern and Central India [21]. The inclusion criterion was any news report published in the specified period pertaining to COVID-19 vaccination including editorials and guest opinion pieces that could potentially generate COVID-19-related vaccine hesitancy.

The two authors read the news report and extracted relevant content and further generated the codes, categories, and themes. The news items were classified into items that had (a) a possible risk of increasing vaccine hesitancy (b) no or minimal risk of increasing vaccine hesitancy. Reports having a potential for increasing vaccine hesitancy in India were considered as those focusing on negative information especially side effects of COVID-19 vaccines (while lacking positive information) and reports that either directly or indirectly through statements of influential social, political, or scientific actors doubted the safety and efficacy of any of the available COVID-19 vaccines while attributing political motives in the government decision for initiating COVID-19 vaccination, without being subject to any fact-check from public sources including the official regulatory and/or apex research body. 

The news-items related with possible risk of increasing vaccine hesitancy were further analyzed based on (A). Content: hesitancy against a specific vaccine, hesitancy related to vaccine safety, hesitancy related to vaccine efficacy, lexicalization (domestic vaccines superior to foreign vaccines), reverse lexicalization (developed world vaccines superior to domestic vaccines). (B). Sources of information: from public health experts, policy experts, medical practitioners, doctor associations, medical journals, and political actors (C) Extent of fact-checking of controversial statements within these reports that were capable of inciting vaccine hesitancy among the readers by the editors. 

## Results

A total of 58 Articles in the Hindi daily (DJ) and 59 articles in the English daily (HT) were identified related to COVID-19 vaccination in India during the period from Jan to Feb 2021 of which there were eight unique newspaper reports after excluding duplicated bilingual entries that met our criterion for possibly contributing to vaccine hesitancy. In addition, we also found another four news items from online digital Indian news sources that also met the same criterion. The reports were grouped into the following two themes, which emerged from the content analysis.

Doubts on the safety and efficacy of locally manufactured vaccines

The decision to provide EUA in clinical trial mode for Covaxin was arrived at in scientific consultation with the Indian Council of Medical Research (ICMR), the apex regulatory body for medical research in India and the SEC of the CDSCO [18]. Multiple opinion pieces criticized the regulatory approval for Covaxin (BBV152), the indigenous COVID-19 vaccine developed for restricted use in clinical trial mode without availability of peer-reviewed phase-3 efficacy data [22-24]. Some researchers, physicians, and journalists apprehended this decision to run the risk of eroding public trust and confidence in COVID-19 vaccines while undermining pandemic control efforts [25,26]. Finally, opposition to Covaxin was also constructed as a ‘political project’ of the government of India that was capable of endangering public health through circulation of statements and opinion pieces by sections of the political establishment, experts, and columnists that found coverage in the mainstream media [27-29].

In contrast to Covaxin, specific opposition to Covishield (ChAdOx1 nCoV-19) vaccine in Indian news sources was rare. One article by a group of public health ethicists demanded a detailed investigation into a few early post-vaccination (with Covishield) deaths among healthcare workers that were stated to be coincidental by the government [30].

Doubts on vaccine requirement considering high SARS-CoV-2 antibody seroprevalence and reduced virus transmission

We found one report that recommended against vaccinating the entire population with COVID-19 vaccines using the conjecture that high levels of SARS-CoV-2 seropositivity were already present in the general population who were likely to have some immune protection. The authors recommended restricting vaccination to vulnerable older adults and those that were seronegative as there was limited availability of vaccines, which in their view should have been prioritized for the vulnerable population groups [31].

A summary assessment of the news reports is reported in Table 1.

**Table 1:** Press Reports with potential for COVID-19 vaccine hesitancy in India (Jan-Feb 2021) DJ: Dainik Jagran; HT: Hindustan Times

S.N.	Title	Date	Content type	Source	Fact-Check
1	Claiming Covaxin as BJP Vaccine; politician surrounded in controversy [32]	DJ 03 Jan 2021, Page 1; HT 02 Jan 2021	Fear of side effects	Politician	Yes, partial. DJ Report condemned politicization of the vaccination process but none in HT report
2	Sparks fly over ‘water’: Covaxin hits out at critics, says trials 200% honest [33]	HT 05 Jan 2021	Doubtful efficacy	Rival vaccine manufacturer, unnamed experts	None. The report mentions that unnamed “experts” had pointed out that the government acted in haste in granting approval to Covaxin
3	Death of vaccine volunteer not related to dose: Bharat Biotech (Covaxin) [34]	DJ 10 Jan 2021, Page 10; HT 10 Jan 2021	Fear of side effects	Manufacturer	Yes, Partial. Statement of the manufacturer reported in detail by both DJ and HT. Family account attributing death of the trial volunteer to intake of the vaccine was also reported by DJ but not in HT.
4	RML resident doctors refuse to take Covaxin (DJ); RML resident doctors raise concerns over Covaxin shots (HT) [35]	DJ 17 Jan 2021, Page 3; HT 17 Jan 2021	Fear of side effects (Covaxin)	Physicians	None. However, DJ but not HT reported that compensation would be provided on occurrence of serious side effects
5	52 have mild side effects, one person serious after vaccination (DJ); India inoculates 220k individuals over two days of Covid-19 vaccination drive (HT) [36]	DJ 17 Jan 2021, Page 3; HT 17 Jan 2021	Fear of side effects	Unnamed public officials	Yes, DJ also reported that minor vaccine related adverse effects were commonplace.
6	580 adverse events reported in three days of vaccination (HT) [37]	HT 19 Jan 2021	Fear of side effects	Spokesperson, Union Health Ministry	Official statement reported, with 3 deaths following vaccination but suspected to be coincidental.
7	Vaccination lagging, govt appeals against hesitancy [38]	HT 20 Jan 2021, Page 1	Fear of side effects	Public Health and Policy Experts	Report emphasizing that vaccination was severely lagging among doctors and nurses. One leading public policy and one public health expert affiliated to the government were quoted encouraging vaccination and dispelling misconceptions regarding vaccination.
8	Senior docs told to take shots to instil confidence [39]	HT 20 Jan 2021, Page 5	Fear of side effects	Senior Doctor	Report focusses on low utilization of Covid-19 vaccines by doctors in some of the city hospitals. Mentions low rate of AEFI reported till date.
9	DCGI’s Covaxin ‘approval’ is political jumla (empty promise). It reinforces idea of Modi’s Atmanirbhar (Self-reliant) Bharat (India) [28]	The Print 5 Jan 2021	Fear of side effects Doubtful efficacy (Covaxin) Reverse Lexicalization	None (unnamed experts)	None. The authors condemn the decision to provide approval for Covaxin prior to completion of Phase-3 trials and insinuates that the decision was influenced politically by the Prime Minister of India. Author claims this could endanger public health in India, and furthermore "undermine India's credibility as a healthcare and pharmaceutical provider". The author dismisses the regulator's claim of the vaccine being safe and makes no reference to the continued safety monitoring conducted by the trial's drug and safety monitoring board as mandated by law.
10	Here's Why I will not take the COVID-19 Vaccine [29]	The Outlook 16 Jan 2021	Fear of side effects Doubtful efficacy Vaccination unnecessary	Self (faculty in preventive and community medicine at a central university)	None. Claims government has secret “agenda and “dangerous game plan” in introducing the vaccine
11	Majority Indians have natural immunity vaccinating entire population can cause great harm [31]	The Print 11 Jan 2021	Vaccination unnecessary (in those with COVID-19 seropositivity)	Self (faculty of Medicine at university in USA)	None. Recommends vaccination for only elderly and avoiding vaccinating the previously infected younger population
12	Nine health workers have died in vaccine rollout. India must disclose status of probe into each case [30]	The Scroll 28 Jan 2021	Fear of side effects (Covishield)	Self (Medical ethicists) and other unnamed experts	Demand for investigation and transparency with making public the findings of AEFI investigation with appropriate compensation (if proved causal).

## Discussion

Suboptimal levels of intention for vaccination and vaccine hesitancy were reported globally in the initial months following introduction of COVID-19 vaccination [40,41]. In India, high levels of vaccine hesitancy weakened public health efforts in controlling the COVID-19 pandemic during the period from Jan to April 2021 and contributed to excess and avoidable deaths during the Delta wave of the pandemic that peaked between April to June 2021. High levels of vaccine hesitancy resulting in wasted doses especially for Covaxin (BBV152) were observed soon after launch of India’s vaccination drive in Jan 2021 [42]. 

Most of the published newspaper reports examined in this study echoed official news sources and views from government health agencies promoting COVID-19 vaccine acceptance and dispelling doubts on concerns regarding vaccine safety. However, multiple news reports also referred to the statements issued by a host of stakeholders including scientists in non-governmental-organizations, columnists, physicians, and opposition politicians condemning regulatory approval for Covaxin (BBV152) without peer-reviewed phase-3 safety and efficacy data, and some also linking the decision to be under duress and for furthering the political and nationalist aspirations of the government of India. These reports were usually not accompanied by any fact checks and this vacillation in contesting the narratives contributing to vaccine hesitancy could be attributed to the schism and lack of consensus in the scientific community regarding this decision. 

However, the ethical validity of the decision in terms of adherence to fundamental bioethical principles was intact when considering this improvisation during an unprecedented pandemic situation. Approval of Covaxin (BBV152) supported the principle of beneficence as it enabled the beneficiary to receive a guaranteed vaccine dose while also being protected through the clause of compensation, akin to participation in a clinical trial. In contrast, a phase-3 trial participant had only half the probability of receiving the actual vaccine dose as against placebo. Furthermore, the SEC also had access to trial data relating to adverse effects following immunization (AEFIs) from the nearly 22,000 trial participants that safeguarded the principle of non-maleficence (CTRI/2020/11/028976). Consequently, the lack of results of the phase-3 trial data in the public domain did not substantially diminish the anticipated vaccine safety because of the absence of any serious AEFI event report that would have required the Data and Safety Monitoring Board (DSMB) to halt the trial. Finally, it may be argued that the decision upheld the principle of distributed justice at a time of absolute vaccine scarcity as it allowed people to have the opportunity to protect themselves by getting a scarce medical resource (vaccine) through an informed choice. Future evidence from phase-3 double-blinded placebo-controlled trials and follow-up epidemiological studies in the community have demonstrated the excellent safety profile and acceptable efficacy of Covaxin (BBV152) [4,43,44].

The then Director General of ICMR alluded to a colonial mindset of some experts that probably influenced their criticism of Covaxin (BBV152) since it was indigenously manufactured [45]. However, in this analysis, we did not detect any significant evidence of a narrative suggesting intrinsic superiority of Western mRNA vaccines as compared to those available in the country although there were references to comparatively stringent regulatory practices informing vaccine development in the developed world as opposed to India [28]. Direct comparisons were also largely superfluous in this period (Jan-April 2021) since doses of the mRNA vaccines were unavailable for developing countries at that point in time, and then prevalent recommendation for sub-zero temperature cold chain requirement rendered it operationally unfeasible in most of the lower-middle-income country locales. Nevertheless, this analysis did not screen social media and television news conversations wherein narratives of partial lexicalization (domestic vaccines as cheaper and more cost-effective compared to foreign vaccines) or reverse lexicalization (foreign vaccines being best in terms of efficacy, safety, and manufacturing standards) were reported [46]. However, with substantial increase in vaccine demand with progression of second wave of the pandemic, criticism accusing the government of India of failure to procure international vaccines to meet domestic needs was rampant [47]. 

A single report from the medical-scientific community expressed concern over the lack of transparent investigation into reports of nine deaths of healthcare workers following Covishield administration that were stated to be coincidental by the government [30]. In contrast, a parallel incident of 23 deaths following administration of mRNA vaccines in the frail elderly reported in Norway in the same period [48] was correctly not associated with allegations of non-transparency suggestive of a reverse lexicalization process wherein Indian drug and vaccine regulatory processes were perceived as lacking trustworthiness compared to developed world standards. In this context, early COVID-19 vaccine confidence in India was rendered a function of the extent of trust in the government (and administration) while vaccine hesitancy correlated with government distrust, a finding supported by empirical survey data [49]. 

The exaggeration of the risks of COVID-19 vaccination or vaccine-related misinformation through any channels of information could negatively impact vaccine acceptance in the general population [7,11,50]. This study is the first in India to have conducted a content analysis to examine print and digital mainstream media reporting that potentially contributed to vaccine hesitancy at the onset of the COVID-19 vaccination drive in the country. Both print and digital mainstream media in India possibly contributed to the politicization of the COVID-19 vaccination process to a variable extent which may have reduced public trust in the vaccine and increased vaccine hesitancy in the months immediately following the introduction of the COVID-19 vaccination in India. Future studies should explore the underpinnings of this phenomenon through in-depth interviews and discussions with multiple stakeholders including scientists affiliated with both public and private institutions, media editors and science journalists and strategize on restricting and overcoming these challenges in future pandemics which may require similar rapid vaccine development and deployment in vulnerable and exposed populations. 

However, there are certain study limitations. First, television and social media conversations on vaccine hesitancy or confidence were not evaluated in this study. Second, articles were not screened in languages other than English or Hindi which limits the generalizability of the findings to mostly that in Northern India. Third, reduced vaccine demand in the populations that were eligible for vaccination was not merely a function of vaccine hesitancy arising from misinformation but also challenges arising from limited vaccine access and availability.

## Conclusions

Concerns about the efficacy and safety of Covaxin (BBV152), the safety of the Covishield vaccine, and questioning the necessity of immunizing all adults with COVID-19 vaccines were observed in media reports in the early stages of the COVID-19 vaccination drive in India until prior to the second wave of the COVID-19 pandemic. Newspaper reports mostly echoed government and administrative sources that allayed the concerns over risk of vaccine-related adverse effects, in contrast to digital media reports which included opinion pieces that were highly critical of the emergency use authorization (EUA) for Covaxin (BBV152) and linked the decision with vaccine nationalism. Furthermore, the reporting on political opposition to Covaxin (BBV152) was usually not associated with fact checks from scientific experts representing the health administration.

The significant vaccine hesitancy at launch of the COVID-19 vaccination drive in India and its association with newspaper and digital media reporting has important lessons in future pandemic preparedness. The EUA for vaccines in clinical trial mode, when necessary, must be accompanied with clear and transparent communication through press releases and press conferences by the government and administration especially with regards to the justification and necessity of the decision, clear explanation of mechanism of safeguarding of public health through monitoring of phase-3 safety data, and the advantage and protection conferred on the vaccinated beneficiaries. The press should avert politicization of the vaccination process when reporting controversial statements from influential public actors with potential for driving vaccine hesitancy by accompanying them with balanced fact-checks from prominent public health and science experts. Finally, the search for scientific consensus must guide drug- and vaccination-related disagreements during pandemic emergencies to maximize public confidence in vaccines.

## References

[1] (2023). Deadly Delta wave stole 2.4 lakh lives in India, ‘similar episodes’ could take place in near term: UN report. https://indianexpress.com/article/india/covid-third-wave-delta-omicron-un-report-7722376/.

[2] Sampathkumar P (2021). Lessons from India's second wave: real world effectiveness of health care worker vaccination. Mayo Clin Proc.

[3] Liu Q, Qin C, Liu M, Liu J (2021). Effectiveness and safety of SARS-CoV-2 vaccine in real-world studies: a systematic review and meta-analysis. Infect Dis Poverty.

[4] Bhatnagar T, Chaudhuri S, Ponnaiah M (2022). Effectiveness of BBV152/Covaxin and AZD1222/Covishield vaccines against severe COVID-19 and B.1.617.2/Delta variant in India, 2021: a multi-centric hospital-based case-control study. Int J Infect Dis.

[5] Sharma P, Basu S, Mishra S, Mundeja N, Charan BS, Singh G, Singh MM (2022). COVID-19 vaccine acceptance and its determinants in the general population of Delhi, India: a state level cross-sectional survey. Cureus.

[6] Chandorkar A, Sudhir S (2023). Braving a Viral Storm: India’s Covid-19 Vaccine Story.

[7] Zhou L, Ampon-Wireko S, Xu X, Quansah PE, Larnyo E (2022). Media attention and vaccine hesitancy: examining the mediating effects of fear of COVID-19 and the moderating role of trust in leadership. PLoS One.

[8] Ahmed S, Rasul ME, Cho J (2022). Social media news use induces COVID-19 vaccine hesitancy through skepticism regarding its efficacy: a longitudinal study from the United States. Front Psychol.

[9] Wang R, Qin C, Du M, Liu Q, Tao L, Liu J (2022). The association between social media use and hesitancy toward COVID-19 vaccine booster shots in China: a web-based cross-sectional survey. Hum Vaccin Immunother.

[10] (2023). How trust in experts and media use affect acceptance of common anti-vaccination claims. https://misinforeview.hks.harvard.edu/article/users-of-social-media-more-likely-to-be-misinformed-about-vaccines/.

[11] Piltch-Loeb R, Savoia E, Goldberg B (2021). Examining the effect of information channel on COVID-19 vaccine acceptance. PLoS One.

[12] Osuagwu UL, Mashige KP, Ovenseri-Ogbomo G (2023). The impact of information sources on COVID-19 vaccine hesitancy and resistance in sub-Saharan Africa. BMC Public Health.

[13] Van Dijk TA (1995). Discourse semantics and ideology. Discourse Soc.

[14] Abbas AH (2022). Politicizing COVID-19 vaccines in the press: a critical discourse analysis. Int J Semiot Law.

[15] Singh K, Verma A, Lakshminarayan M (2022). India's efforts to achieve 1.5 billion COVID-19 vaccinations: a narrative review. Osong Public Health Res Perspect.

[16] Jeewandara C, Kamaladasa A, Pushpakumara PD (2021). Immune responses to a single dose of the AZD1222/Covishield vaccine in health care workers. Nat Commun.

[17] (2023). Press Statement by the Drugs Controller General of India (DCGI) on Restricted Emergency approval of COVID-19 virus vaccine. Jan.

[18] (2023). The Subject Expert Committee (SEC) of Central Drugs Standards Control Organisation (CDSCO) makes recommendations in respect of Accelerated Approval Process request of M/s Serum Institute of India and M/s Bharat Biotech International Ltd as well as about Phase-III Trials of M/s Cadila Healthcare Ltd. https://pib.gov.in/PressReleasePage.aspx?PRID=1685678.

[19] Ella R, Vadrevu KM, Jogdand H (2021). Safety and immunogenicity of an inactivated SARS-CoV-2 vaccine, BBV152: a double-blind, randomised, phase 1 trial. Lancet Infect Dis.

[20] (2023). Ministry of Health and Family Welfare: Frequently asked questions. https://www.mohfw.gov.in/covid_vaccination/vaccination/faqs.html.

[21] (2023). Audit Bureau of Circulation. http://www.auditbureau.org/files/JD2018%20Highest%20Circulated%20(language%20wise).pdf.

[22] Mohapatra PR, Mishra B (2021). Regulatory approval of COVID-19 vaccine for restricted use in clinical trial mode. Lancet Infect Dis.

[23] Biswas S (2023). Covaxin: What was the rush to approve India's homegrown vaccine?. https://www.bbc.com/news/world-asia-india-55534902.

[24] (2023). Scientists criticize ‘rushed' approval of Indian COVID-19 vaccine without efficacy data. https://www.science.org/content/article/scientists-criticize-rushed-approval-indian-covid-19-vaccine-without-efficacy-data.

[25] (2023). Coronavirus | I would not take the vaccine without efficacy data: Gagandeep Kang. https://www.thehindu.com/sci-tech/health/i-would-not-take-the-vaccine-without-efficacy-data-gagandeep-kang/article33527186.ece.

[26] (2023). "Will Prefer Covishield Over Covaxin": Delhi Hospital Doctors Concerned. https://www.ndtv.com/india-news/give-us-vaccine-that-cleared-all-trials-not-other-one-delhi-rml-hospital-doctors-2353417.

[27] (2023). After Congress’ Jairam & Tharoor, now Milind Deora questions ‘speedy’ approval for Covaxin. https://theprint.in/politics/after-congress-jairam-tharoor-now-milind-deora-questions-speedy-approval-for-covaxin/579288/.

[28] (2023). DCGI’s Covaxin ‘approval’ is political jumla. It reinforces idea of Modi’s Atmanirbhar Bharat. https://theprint.in/opinion/dcgis-covaxin-approval-is-political-jumla-it-reinforces-idea-of-modis-atmanirbhar-bharat/579337/.

[29] (2023). Here's Why I Will Not Take The Covid-19 Vaccine: JNU Professor Vikas Bajpai. https://www.outlookindia.com/website/story/opinion-why-i-will-not-take-the-covid-19-vaccine/370695.

[30] (2023). Nine health workers have died in vaccine rollout. India must disclose status of probe into each case. https://scroll.in/article/985273/nine-health-workers-have-died-in-vaccine-rollout-india-must-disclose-status-of-probe-into-each-case.

[31] (2023). Majority Indians have natural immunity. Vaccinating entire population can cause great harm. https://theprint.in/opinion/majority-indians-have-natural-immunity-vaccinating-entire-population-can-cause-great-harm/582174/..

[32] (2023). ‘How can I trust BJP’s Covid-19 vaccine’. https://www.hindustantimes.com/india-news/how-can-i-trust-bjp-s-covid-19-vaccine-akhilesh-yadav-won-t-get-vaccinated-for-now/story-wymck18etFE26C7tqyEuZM.html.

[33] (2023). Sparks fly over ‘water’: Covaxin hits out at critics, says trials 200% honest. https://www.hindustantimes.com/india-news/phase-3-covaxin-data-in-feb-or-march-bharat-biotech/story-QetWiqSy1ATBaKooOjWHsN.html.

[34] (2023). Death of vaccine volunteer not related to dose: Bharat biotech. https://www.hindustantimes.com/india-news/death-of-vaccine-volunteer-not-related-to-dose-bharat-biotech-101610221817475.html.

[35] (2023). RML resident docs raise concerns over Covaxin shots. https://www.hindustantimes.com/cities/others/rmlresident-docs-raise-concerns-over-covaxin-shots-101610819511114.html.

[36] (2023). India inoculates 220k individuals over two days of Covid-19 vaccination drive. https://www.hindustantimes.com/india-news/india-vaccinates-224-301-individuals-over-two-days-of-covid-19-vaccination-drive-101610909794711.html.

[37] (2023). 580 adverse events reported in three days of vaccination. https://www.hindustantimes.com/india-news/580-adverse-events-reported-in-three-days-of-vaccination-101611000733824.html.

[38] (2023). Vaccination lagging, govt appeals against hesitancy. https://www.hindustantimes.com/india-news/vaccination-lagging-govt-appeals-against-hesitancy-101611077863927.html.

[39] (2023). Senior docs told to get vaccinated to instil confidence. https://www.hindustantimes.com/india-news/senior-docs-told-to-get-vaccinated-to-instil-confidence-101611079986044.html.

[40] Sallam M (2021). COVID-19 vaccine hesitancy worldwide: a concise systematic review of vaccine acceptance rates. Vaccines (Basel).

[41] Noushad M, Rastam S, Nassani MZ (2021). A global survey of COVID-19 vaccine acceptance among healthcare workers. Front Public Health.

[42] (2023). Covid-19 vaccine doses wasted in more states over hesitancy. https://www.hindustantimes.com/india-news/covid19-vaccine-doses-wasted-in-more-states-over-hesitancy-101611182030965.html.

[43] Parida SP, Sahu DP, Singh AK (2022). Adverse events following immunization of COVID-19 (Covaxin) vaccine at a tertiary care center of India. J Med Virol.

[44] Ahmed TI, Rishi S, Irshad S (2022). Inactivated vaccine Covaxin/BBV152: a systematic review. Front Immunol.

[45] Bhargava B (2021). Going Viral: Making of Covaxin - The Inside Story.

[46] Chatterjee N, Mahmood Z, Marcussen E (2021). Politics of vaccine nationalism in India: global and domestic implications. Forum Dev Studies.

[47] (2023). Myths & Facts on India’s Vaccination Process. https://pib.gov.in/PressReleasePage.aspx?PRID=1722078.

[48] Torjesen I (2021). Covid-19: Norway investigates 23 deaths in frail elderly patients after vaccination. BMJ.

[49] (2023). Most BJP supporters want to take vaccines, Congress supporters not far behind. https://www.livemint.com/politics/news/most-bjp-supporters-want-to-take-vaccines-congress-supporters-not-far-behind-11610115072702.html.

[50] Jennings W, Stoker G, Bunting H (2021). Lack of trust, conspiracy beliefs, and social media use predict COVID-19 vaccine hesitancy. Vaccines (Basel).

